# Control over the Percentage, Shape and Size of the Graphite Particles in Martensitic White Castings Alloyed with Cr, Nb and Mg

**DOI:** 10.3390/ma12010185

**Published:** 2019-01-08

**Authors:** Alberto Cofiño-Villar, Florentino Alvarez-Antolin, Juan Asensio-Lozano, Maria Garcia-Garcia

**Affiliations:** Materials Pro Group, Departamento de Ciencia de los Materiales e Ingeniería Metalúrgica, Universidad de Oviedo, Independencia 13, 33004 Oviedo, Spain; UO229780@uniovi.es (A.C.-V.); jasensio@uniovi.es (J.A.-L.); magarc@uniovi.es (M.G.-G.)

**Keywords:** work roll, white cast iron, graphite, inoculation, M_3_C, MC, nodularity

## Abstract

This paper presents the results obtained regarding the control by manufacturers of the percentage, shape, and size of the precipitated graphite in the working layer of duplex work-rolls used in hot strip mill finishing stands. This working layer is manufactured in a martensitic white cast iron alloyed with Cr and Nb to promote the precipitation of M_3_C and MC carbides, which provide a high wear resistance. The thermal cycling behavior of this layer also has a decisive influence on its service life. In this context, the percentage of graphite and its morphology play a very important role against said thermal cycling. With the aim of studying their effect on the sphericity of graphite, the analyzed industrial manufacturing factors worth highlighting include the liquidus temperature, the %Si, the use of an FeSi inoculant with traces of Lanthanum, inoculation with different amounts of FeB and SiCaMn, and the addition of Mg. At the periphery of the working layer, it was found that the use of the FeSi inoculant with traces of La promoted an increase in the density of counts of graphite, and that inoculation with FeB and the addition of 0.02% Mg diminished the nodularity of the graphite. Furthermore, throughout the entire thickness of the working layer, an increase in the amount of SiCaMn of up to 0.6 kg/T produced an increase in the size of the graphite particles and a marked improvement in their nodularity.

## 1. Introduction

The failure mechanisms that arise in the surface of indefinite chill double-poured (ICDP) work rolls used in the finishing stands of hot strip mills involve phenomena of plastic deformation, abrasive wear, and cracking resulting from mechanical or thermal stresses [1]. These work rolls are manufactured by means of vertical centrifugal casting. The outer working layer is a martensitic white cast iron with the presence of graphite particles, while the core is a grey cast iron with a pearlitic matrix and dispersed spheroidal graphite. Its main alloying elements are Ni and Cr. The chemical composition may include Nb and Mo so as to improve the wear resistance of the working layer. The Nb forms carbides of the MC type with a hardness close to 2400 HV [2], while the Cr forms carbides of the M_3_C type [3], with a hardness close to 1200 HV [3], and the Ni and the Mo increase the hardenability of the material [4]. Thus, its microstructure will mainly be made up of proeutectic austenite, eutectic austenite, MC and M_3_C carbides, and graphite particles [5]. The austenite will be partially converted to martensite during air cooling after quenching at 1000 °C. During the rolling process, the working layer is heated on entering into contact with the sheet steel to be rolled. This heating is counteracted by spraying with water as the materials enters and exits the rolling pass [6]. During the initial stages in the finishing stands, the surface of the work rolls can reach over 500 °C, subsequently cooling down to 50 to 80 °C during water cooling [7], thereby producing thermal fatigue cycles. During the rolling pass, the work roll, likewise, undergoes compression forces that oppose thermal expansion. Hence, the service life of these rolls depends not only on their wear resistance but also on their fatigue behavior versus cyclic thermal and mechanical stresses [8]. In this context, in addition to the presence of carbides and their network distribution, the percentage of graphite and its morphology also play a very important role against said thermal cycling. Although crack nucleation is predominant in carbides, the subsequent progression of cracks may be favored by laminar graphite morphologies [9,10]. However, thermal conductivity is improved by this morphology compared to compact and spheroidal morphologies [11]. Thus, the laminar morphology of graphite improves the evacuation of heat, but favors the progression of the possible cracks that are generated in the carbide network during the rolling process. In contrast, a spheroidal geometry reduces the evacuation of heat, but constitutes a barrier to the progression of said cracks. Furthermore, graphite performs dry lubrication functions [12], reducing the coefficient of friction between the work roll and the sheet steel to be rolled [13]. One of the quality indicators established for the working layer of these rolls is the volume fraction of graphite. Optimal quality is achieved when this percentage falls within the 2 to 5% range [14]. Hence, one of the objectives of manufacturers and users of this type of work roll should be to control both the volume fraction of graphite and its geometry, in addition to knowing how the different variables of the manufacturing process affect these parameters. This paper presents a research methodology aimed at achieving these objectives in white cast irons alloyed with Ni, Cr, Mo, and Nb. To this end, a fractional Design of Experiments (DOE) is applied to identify those Factors in the manufacturing process that have a significant influence on the percentage of precipitated graphite and its geometry. The analyzed manufacturing Factors were: the use of inoculants based on FeSi alloys that include traces of lanthanum; inoculation with different percentages of Mg, FeB, and SiCaMn; the liquidus lemperature, and the percentage of Si. Both the volume fraction and the number of counts of graphite increase with increasing equivalent carbon (EC), while Mg favors the precipitation of graphite with a spheroidal morphology [15,16]. There are many theories that justify the possible spheroidal growth of the graphite. It should be noted that, one of them, which has been proven on iron–carbon–silicon alloys with no sulphur contamination, vacuum caste, and without any presence known of any spheroidizing element (Mg, Ce, RE, etc.). This theory is based on the fact that graphite–liquid surface energy is higher in spheroidal graphite than in flake graphite, so that the crystalline growth of graphite will “curve” to reduce the free surface energy [17], or to decrease the energy–volume ratio [18]. The carbon equivalent implicitly includes factors, such as the %Si and the liquidus temperature, both of which are analyzed in this paper. The composition of FeSi alloys includes elements such as Ca, Al, and Ba, which, due to their high affinity with O, precipitate forming small oxide particles that can act as agents for the nucleation of graphite [19]. The use of inoculants with high percentages of Ca may promote a decrease in the grain size of the primary austenite and discontinuity of the carbide network [20]. La could increase the number of counts of graphite [21] and promote a finer structure of the carbide network [22]. B may combine with the N dissolved in the molten metal forming particles of BN that can act as heterogeneous nucleating agents of graphite [23]. B can also form carbides, thereby improving the wear resistance of the material [24,25]. Nb tends to reduce the eutectic cell, which could result in graphite of smaller size that is more uniformly distributed [19,26]. The formation of Nb carbides in the liquid phase could promote heterogeneous nucleation of graphite [27]. These carbides can also increase the hardness of the resulting material and improve its wear resistance [26]. The study was carried out on an industrial scale, casting eight work rolls with a diameter of 700 mm, a length of between 1800 and 2000 mm, and a thickness of the working layer of 50 mm.

## 2. Materials and Methods

Table 1 shows the standard chemical composition range for these working layers. The working layer was cast first and then the core was subsequently cast in two stages. In the first stage, an intermediate layer was cast aimed at ensuring optimum binding with the working layer. The remainder of the core was then cast. The working layer was smelted in a medium frequency induction furnace. The inoculants were placed on the bottom of the ladle when “bleeding” the molten iron. This bleeding occurred at a temperature of 1420 °C. The casting was carried out at a temperature of around 60 °C above the liquidus temperature. Demoulding took place 4 or 5 days after casting. After quenching at 1000 °C with air cooling, the roll was subjected to tempering at 400 °C.

The experimental procedure employed was based on the Design of Experiments statistical technique [28]. In this case, performing a total of 8 experiments, 6 industrial manufacturing Factors were analyzed, each Factor varying between 2 levels. Table 2 shows the analyzed Factors and levels, while Table 3 displays the Array of Experiments, together with the confounding pattern. These same manufacturing factors were studied in a previous work of the authors, where the resistance of the working layer against mechanical stresses was analyzed, correlating the results with the volume fraction of precipitated carbides [29]. The set of generators associated with this array of experiments is D = AB, E = AC, and F = BC, whose resulting relation definition is I = ABD = ACD = BCF, where I is a column formed only by some (+1) [28]. The resolution of this design is III, i.e., the main effects are confounded with the interactions of two Factors [28]. Table 4 shows the chemical composition of the inoculants employed in this study. Table 5 shows the main casting parameters of the 8 experiments (8 work rolls).

For the analysis of the volume fraction and morphology of the precipitated graphite, samples were obtained from two regions of the working layer. One of them, denominated Zone α, which comprised a thickness of 15 mm from the periphery of said working layer, and another, denominated Zone *β*, which comprised another 15 mm of thickness from a distance of 25 mm from the periphery up to a distance of 40 mm. The variables used as “responses” to characterize the graphite particles were:

- The volume fraction of graphite, denoted as *Vv*.
- The number of counts per mm^2^ of graphite, denoted by *N_A_*.
- The mean Feret diameter, denoted by *F_mean_*.
- The roundness parameter, denoted by *R* and defined as $\frac{{\left[perimeter\right]}^{2}}{4\pi \left(area\right)}$. This parameter defines a perfect nodule when its value is between 1 and 1.4.
- The percentage of counts of graphite with a roundness parameter equal to or less than 1.4.

These responses were determined using the Image ProPlus software (version 4.5.0.29) (Media Cybernetics, Rockville, MD, USA), together with its Materials-Pro analysis module. For this purpose, 10 micrographs were randomly obtained at 100× in each experiment and in each region (Zones *α* and *β*). The metallographic samples were in the polished state, without etching with a chemical reagent. The optical microscope employed was a NIKON Epiphot 200 (Nikon, Tokyo, Japan), the images being obtained using the Omnimet Enterprise Image Analysis System. A JEOL JSM-5600 scanning electron microscope (JEOL, Nieuw-Vennep, The Netherlands), equipped with the characteristic energy dispersive X-ray (EDX, JEOL, Nieuw-Vennep, The Netherlands) microanalysis system, was used to obtain representative micrographs of the general microstructure. A CAMEBAX SX-100 electronic probe (CAMECA, Gennevilliers, France), equipped with BSE detectors (CAMECA, Gennevilliers, France), was used to obtain images of backscattered electrons. In these latter two cases, the samples were in the polished state and were etched with the chemical reagent Nital 4.

## 3. Results

**Figure 1:**
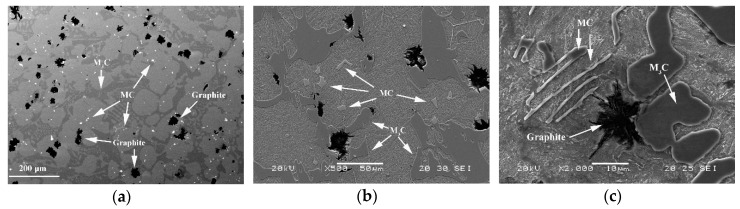
General microstructure of alloys of this type: (**a**) Micrograph obtained using an electronic microprobe, corresponding to Zone *β* in Experiment 2; (**b**) Micrograph obtained using a scanning electron microscope, corresponding to Zone *β* in Experiment 3; (**c**) Micrograph obtained using a scanning electron microscope, corresponding to Zone *α* in Experiment 6.

Figure 1 shows the general microstructure of alloys of this type. The presence of M_3_C and MC carbides and dispersed graphite in an austenitic matrix, which has been transformed into Martensite during its air cooling, can be appreciated. Figure 1a corresponds to a sample from Experiment 2 in an inner region of the working layer (Zone *β*), Figure 1b corresponds to a sample from Experiment 3 in an inner region of the working layer (Zone *β*), and Figure 1c corresponds to a sample from Experiment 6 in an outer region of the working layer (Zone *α*).

**Figure 2:**
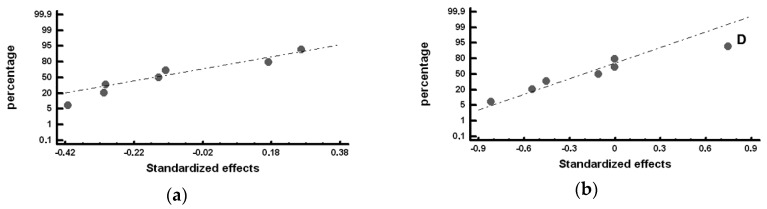
Standardized effects on the volume fraction of graphite on a normal probability plot: (**a**) Factors with a significant effect on the outermost region of the working layer (Zone *α*); (**b**) Factors with a significant effect on the inner region of the working layer (Zone *β*).

**Figure 3:**
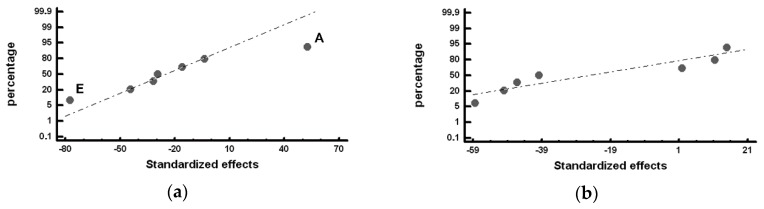
Standardized effects on the number of counts per mm^2^ of graphite, *N_A_*, on a normal probability plot: (**a**) Factors with a significant effect on the outermost region of the working layer (Zone *α*); (**b**). Factors with a significant effect on the inner region of the working layer (Zone *β*).

**Figure 4:**
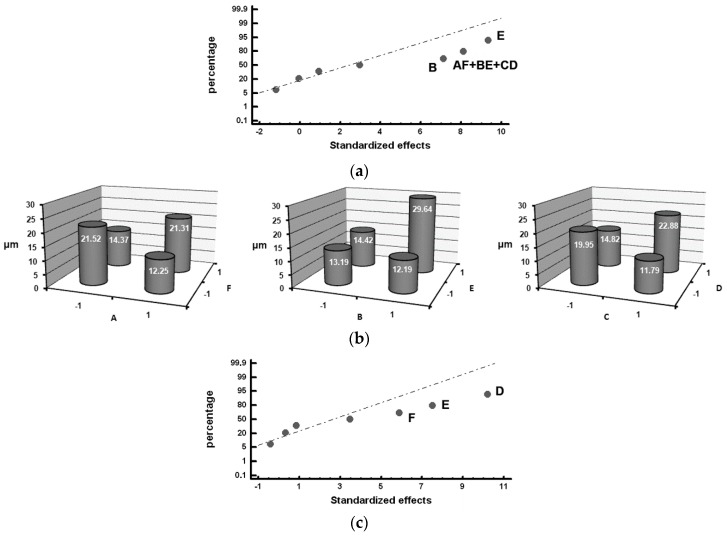
Standardized effects on the mean Feret diameter, *F_mean_*, on a normal probability plot: (**a**) Factors with a significant effect on the outermost region of the working layer (Zone *α*); (**b**) Detailed analysis of Interaction AF + BE + CD, which has a significant effect; (**c**) Factors with a significant effect on the inner region of the working layer (Zone *β*).

**Figure 5:**
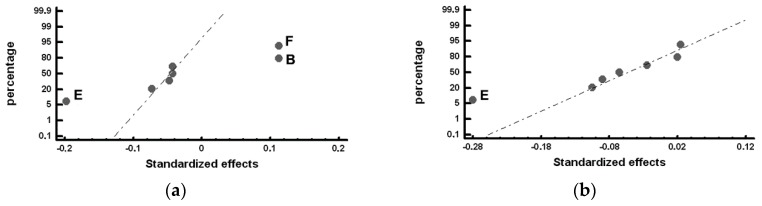
Standardized effects on the Roundness parameter, *R*, on a normal probability plot: (**a**) Factors with a significant effect on the outermost region of the working layer (Zone *α*); (**b**) Factors with a significant effect on the inner region of the working layer (Zone *β*).

**Figure 6:**
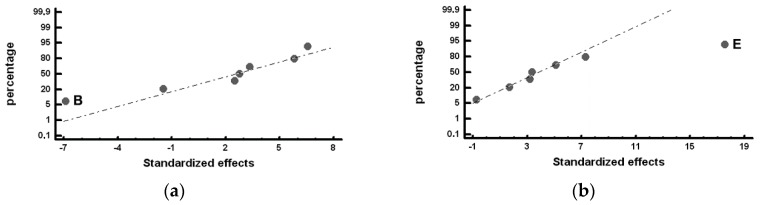
Standardized effects on the percentage of graphite with a nodular morphology, on a normal probability plot: (**a**) Factors with a significant effect on the outermost region of the working layer (Zone *α*); (**b**) Factors with a significant effect on the inner region of the working layer (Zone *β*).

The standardized effects were compared on a normal probability plot using the Statgraphics Plus (version 5.1) program (Statgraphics Technologies, The Plain, VA, USA). The standardized effect is the ratio between the difference in the value of the response and its mean and standard deviation. This represents not only whether the value of the variable is above or below the mean but also how far it deviates from it. Those standardized effects that deviate from the straight line towards the ends on the normal probability plot are significant. Those that deviate to the left indicate that the value of the response increases at their −1 level and, analogously, while those that deviate to the right indicate that the value of the response increases at their +1 level [28]. Table 6, Table 7, Table 8 and Table 9 show the mean values obtained for the studied responses and the standardized effects corresponding to the Factors and Interactions indicated in the column denominated “Confounding Pattern”. The first row of this column refers to the mean value obtained for each of the analyzed responses considering the eight experiments. Figure 2, Figure 3, Figure 4, Figure 5 and Figure 6 show the representation of the standardized effects on a normal probabilistic plot for the analyzed responses.

Figure 2 shows the standardized effects on the volume fraction of graphite on a normal probability plot. Figure 2a shows that, in the outermost region of the working layer (Zone *α*), none of the analyzed Factors has a significant effect on the volume fraction of graphite. However, Figure 2b shows that Factor D (%Si) has a significant effect on the volume fraction of graphite in the inner region of the working layer (Zone *β*): situating this Factor at its +1 level (1.1–1.15 %Si) leads to an increase in the volume fraction of precipitated graphite.

Figure 3 shows the Factors with a significant effect on the number of counts of graphite per mm^2^, *N_A_*. Figure 3a shows that Factor A (inoculation with FeSi-La) and Factor E (inoculation with SiCaMn) have a significant effect on *N_A_* in the outermost region of the working layer: inoculation with FeSi-La and a low level of SiCaMn favor an increase in this variable. However, Figure 3b shows that none of the analyzed Factors has a significant effect on *N_A_* in the inner region.

Figure 4 shows the Factors with a significant effect on the size of the counts of graphite, represented by the mean Feret diameter (*F_mean_*). In Figure 4a, it can be observed that Factor E (inoculation with SiCaMn) and Factor B (addition of FeB) significantly influence this variable in Zone *α* of the working layer: both Factors at their +1 level increase *F_mean_*. It, hence, follows that if Factor E at its −1 level increases the number of counts of graphite, it is because when it is placed at this level, it also reduces the size of said graphite. Interaction AF + BE + CD is also found to have a significant effect. Figure 4b provides a detailed analysis of this interaction, in which it is verified that it is interaction BE that has a significant effect, increasing the variable *F_mean_* when both Factors are at their +1 level. In Figure 4c, it can be observed that Factor F (%Mg), Factor E (SiCaMn), and Factor D (%Si) have a significant effect on *F_mean_* in the inner region of the working layer (Zone *β*): placing these Factors at their +1 level produces an increase in the value of this variable.

Figure 5 shows the Factors with a significant effect on the roundness parameter of the particles of graphite. Figure 5a shows the significant effect of Factor E (SiCaMn), Factor B (FeB), and Factor F (%Mg) in Zone *α* of the working layer: if these Factors are respectively situated at their −1, +1, +1 levels, this parameter increases and the nodularity of the graphite decreases. The deleterious effect of the %Mg on the nodularity of the graphite is worth noting. This result could be justified by the fact that the residual Mg associated with the Mg added at Level +1 (0.02%) is insufficient to achieve a nodular geometry, promoting a “star-shaped” geometry that results in high roundness parameters [30]. Several graphite particles with this geometry can be observed in Figure 1a. Figure 5b shows the significant effect of Factor E (SiCaMn) in Zone *β* of the working layer. As occurred in Zone *α* of the working layer, if this Factor is situated at its −1 level, the roundness parameter increases. The addition of Mg does not have a significant effect in this region.

Furthermore, Figure 6 shows the Factors with a significant effect on the % graphite with a nodular geometry, i.e., with a roundness parameter below 1.4. In Zone *α* of the working layer, Factor B (FeB) is significant: To increase the % nodular graphite, this Factor must be placed at its −1 Level; see Figure 6a. Similarly, in Zone *β* of the working layer, the effect of Factor E (SiCaMn) is found to be significant: placing this Factor at its +1 level will increase the percent of particles of graphite with a spheroidal morphology; see Figure 6b.

## 4. Conclusions

This paper presents a research method for manufacturers and users of duplex work rolls used in hot strip mill finishing stands whose working layers is manufactured in Ni-hard cast iron alloyed with Nb and Mg that enables them to control the volume fraction and morphology of precipitated graphite. As to the manufacturing Factors and their Levels analyzed in this paper, it is concluded that:

1. Inoculation with SiCaMn has a significant effect on the shape and size of the precipitated graphite. An increase of 0.3 to 0.6 kg/T will lead to:
  - an increase in the size of the precipitated graphite, and
  - an improvement in the nodularity of the graphite.
2. Inoculation with FeB has a significant effect on the shape and size of the precipitated graphite in the outermost region of the working layer. An increase of 3 to 6 kg/T will lead to:
  - an increase in the size of the precipitated graphite,
  - a deterioration in the nodularity of the graphite, and
  - a reduction in the percentage of graphite with a nodular morphology.
3. In the outermost region of the working layer in contact with the sheet steel to be rolled:
  - treatment with Mg, up to values of around 0.02%, has a deleterious effect on the nodularity of the graphite.
  - inoculation with FeSiLa produces an increase in the density of graphite nodules.

## Figures and Tables

**Table 1:** Chemical composition range of the working layer.

C	Si	Mn	Ni	Cr	Nb	Mo
3.2–3.4	0.9–1.0	0.8–1.0	4.4–4.6	1.7–1.8	0.65–0.75	0.25

**Table 2:** Factors and Levels analyzed in the Design of Experiments (DOE).

Factors		Levels	
Code	Metallurgical Parameter Correspondence	Level −1	Level +1
A	FeSi-La (Kg/T)	0	2.7
B	FeB (Kg/T)	3	6
C	Liquidus Temperature (°C)	1250–1255	1270–1275
D	Si (%)	0.8–0.85	1.1–1.15
E	SiCaMn (Kg/T)	0.3	0.6
F	Mg (%)	0	0.02

**Table 3:** Array of Experiments.

No.	A	B	C	D	E	F	Confounding Pattern
1	−1	−1	−1	1	1	1	A + BD + CE
2	1	−1	−1	−1	−1	1	B + AD + CF
3	−1	1	−1	−1	1	−1	C + AE + BF
4	1	1	−1	1	−1	−1	D + AB + EF
5	−1	−1	1	1	−1	−1	E + AC + DF
6	1	−1	1	−1	1	−1	F + BC + DE
7	−1	1	1	−1	−1	1	AF + BE + CD
8	1	1	1	1	1	1	

**Table 4:** Chemical composition of the inoculants used (% wt.).

Inoculants	Si	Ca	Al	Mn	Ti	Ba	C	Bi	S	P	B	La	Fe
FeSi-La	66.0	2.5	0.8	---	---	0.3	---	0.3	---	---	---	0.8	rem.
FeMn	2.0	---	---	69.4	---		5.8		0.014	0.130	---		rem.
SiCaMn	58.3	16.4	1.1	14.8	0.030		0.6		0.030	0.030	---		rem.
FeB	0.4	---	---	---	---		0.3		---	---	17.9		rem.

**Table 5:** Casting parameters in each experiment.

Casting Parameters	Units	Experiment Number							
		1	2	3	4	5	6	7	8
C	%	3.35	3.46	3.40	3.28	2.94	3.04	3.02	3.04
Si	%	1.13	0.88	0.87	1.18	1.16	0.89	0.87	1.15
Mn	%	0.77	0.78	0.79	0.77	0.79	0.83	0.80	0.82
Ni	%	4.44	4.33	4.32	4.38	4.59	4.16	4.62	4.65
Cr	%	1.68	1.68	1.71	1.64	1.65	1.71	1.68	1.71
Mo	%	0.26	0.25	0.25	0.24	0.25	0.25	0.26	0.26
Mg	%	0.0024	0.0018	-	-	-	-	0.0022	0.0028
B	%	0.032	0.033	0.071	0.075	0.038	0.041	0.070	0.071
Nb	%	0.64	0.72	0.68	0.61	0.74	0.75	0.73	0.61
Liquidus Temperature	°C	1252	1254	1253	1250	1273	1272	1272	1270

**Table 6:** Average values and standardized effects for the volume fraction of precipitated graphite (*V_V_*).

Experiment	Zone *α*		Zone *β*		Confounding Pattern
	Values	Effect	Values	Effect	
1	1.82	2.149	3.42	2.901	**Mean**
2	2.71	0.268	2.22	−0.003	A + BC + CE
3	2.32	−0.128	2.93	−0.108	B + AD + CF
4	2.57	−0.413	4.13	−0.548	C + AE + BF
5	2.19	−0.148	3.24	0.748	D + AB + EF
6	2.13	−0.303	2.94	−0.003	E + AC + DF
7	1.73	−0.308	2.02	−0.818	F + BC + DE
8	1.72	0.173	2.31	−0.453	AF + BE + CD

**Table 7:** Mean values and standardized effects for the number of count per mm^2^ of graphite (*N_A_*).

Experiment	Zone *α*		Zone *β*		Confounding Pattern
	Values	Effect	Values	Effect	
1	26.77	90.3	43.07	96.103	**Mean**
2	173.35	52.685	159.36	11.5	A + BC + CE
3	26.06	−31.76	156.10	1.885	B + AD + CF
4	142.36	−3.67	142.49	−58.305	C + AE + BF
5	88.56	−44.345	85.75	−50.0	D + AB + EF
6	136.04	−78.755	92.46	−39.84	E + AC + DF
7	114.44	−15.91	76.49	−46.195	F + BC + DE
8	14.82	−29.205	13.10	14.95	AF + BE + CD

**Table 8:** Mean values and standardized effects for the mean Feret diameter (*F_mean_*).

Experiment	Zone *α*		Zone *β*		Confounding Pattern
	Values	Effect	Values	Effect	
1	17	17.361	28.83	19.146	**Mean**
2	11.61	−1.173	11.56	0.863	A + BC + CE
3	28.29	7.118	12.63	0.328	B + AD + CF
4	12.65	−0.053	16.61	3.478	C + AE + BF
5	14.76	2.983	18.49	10.218	D + AB + EF
6	11.84	9.343	17.05	7.508	E + AC + DF
7	11.74	0.953	14.91	5.903	F + BC + DE
8	31	8.108	33.09	−0.408	AF + BE + CD

**Table 9:** Mean values and standardized effects for the Roundness parameter (*R*).

Experiment	Zone *α*		Zone *β*		Confounding Pattern
	Values	Effect	Values	Effect	
1	1.75	1.789	1.75	1.81	**Mean**
2	1.87	−0.048	2	−0.025	A + BC + CE
3	1.72	0.113	1.72	−0.09	B + AD + CF
4	1.90	−0.043	1.98	−0.105	C + AE + BF
5	1.72	−0.043	2.01	0.025	D + AB + EF
6	1.59	−0.198	1.66	−0.28	E + AC + DF
7	2.06	0.113	1.81	−0.065	F + BC + DE
8	1.70	−0.073	1.55	0.02	AF + BE + CD

## References

[1] Nilsson M., Olsson M. (2013). An investigation of worn work roll materials used in the finishing stands of the hot strip mill for steel rolling. Proc. Inst. Mech. Eng. Part J J. Eng. Tribol..

[2] Bedolla-Jacuinde A. (2001). Microstructure of vanadium-, niobium- and titanium-alloyed high-chromium white cast irons. Int. J. Cast Met. Res..

[3] Pero-Sanz J.A. (1994). Fundiciones Férreas.

[4] Pero-Sanz J.A. (2000). Ciencia e Ingeniería de Materiales.

[5] Bravo S.V., Yamamoto K., Miyahara H., Ogi K. Control of carbides and graphite in Ni-hard type cast iron for hot strip mills. Proceedings of the Pricm 6: Sixth Pacific Rim International Conference on Advanced Materials and Processing.

[6] Guerrero M.P., Pérez A., Colás R. Heat Transfer to the work rolls during hot rolling of steel. Proceedings of the Rolls 2000 International Convention Centre Birmingham.

[7] Belzunce F.J., Ziadi A., Rodriguez C. (2004). Structural integrity of hot strip mill rolling rolls. Eng. Fail. Anal..

[8] Noda N.A., Hu K.J., Sano Y., Ono K., Hosokawa Y. (2016). Residual Stress Simulation for Hot Strip Bimetallic Roll during Quenching. Steel Res. Int..

[9] Tercelj M., Fajfar P., Godec M., Kugler G. (2017). Characteristics of the thermal fatigue resistance for 3.1C, 0-8Si, 0.9Mn, 1.7Cr, 4.5Ni and 0-3Mo ICDP cast iron roll at 600 degrees C. Mater. Tehnol..

[10] Bombac D., Kugler G., Markoli B., Tercelj M. (2017). Hot work roller surface layer degradation progress during thermal fatigue in the temperature range 500–700 degrees C. Int. J. Fatigue.

[11] Holmgren D., Dioszegi A., Svensson I.L. (2007). Effects of nodularity on thermal conductivity of cast iron. Int. J. Cast Met. Res..

[12] Sergio V., Ishikawa S., Yamamoto K., Miyahara H., Ogi K., Kamimiyada K. (2008). Control of graphite formation in solidification of white cast iron. Int. J. Cast Met. Res..

[13] Gowda D., Kumar D.C., Sandeep G.M., Parthasarathy A., Chandrashekar S. (2018). Tribological Characterization of Centrifugally Cast Graphite Cast Iron under Dry and Wet conditions. Mater. Today Proc..

[14] Valek T., Hampl J. (2011). Prediction of Metallurgic Quality of ICDP Material before Tapping. Phys. Procedia.

[15] Song J.M., Lui T.S., Chen L.H. (1999). Effect of carbon equivalent and spheroidizer addition on the morphology of strip cast white cast iron plate. Int. J. Cast Met. Res..

[16] Konig M., Wessen M. (2010). Influence of alloying elements on microstructure and mechanical properties of CGI. Int. J. Cast Met. Res..

[17] Geilenberg H., Merchant H.D. (1968). A critical Review of the Crystalization of Graphite from Metallic Solutions after the “Surface Tension Theory”. Recent Reaearch on Cast Iron.

[18] Sadocha J.P., Gruzleski J.E., Lux B. (1975). The Mechanism of Graphite Spheroid Formation in Pure Fe-C-Si Alloys. The Metallurgy of Cast Iron.

[19] Strande K., Tiedje N., Chen M. (2017). A Contribution to the Understanding of the Combined Effect of Nitrogen and Boron in Grey Cast Iron. Int. J. Metalcast..

[20] Dun X.L., Liu K.P., Liu H.S., Lai J.P., Fu X.H., Zhou J. (2011). Effect of multicomponent modifier on microstructure and mechanical properties of high Ni-Cr-Mo cast iron. Mater. Sci. Technol..

[21] Onsoien M.I., Skaland T., Grong O. (1999). Mechanisms of graphite formation in ductile cast iron containing cerium and lanthanum. Int. J. Cast Met. Res..

[22] Hamidzadeh M.A., Meratian M., Saatchi A. (2013). Effect of cerium and lanthanum on the microstructure and mechanical properties of AISI D2 tool steel. Mater. Sci. Eng. Struct. Mater. Prop. Microstruct. Process..

[23] Eppich R. (2006). Cast Iron Alloy Containing Boron. U.S. Patent.

[24] Col M., Koc F.G., Oktem H., Kir D. (2016). The role of boron content in high alloy white cast iron (Ni-Hard 4) on microstructure, mechanical properties and wear resistance. Wear.

[25] Tasgin Y., Kaplan M., Yaz M. (2009). Investigation of effects of boron additives and heat treatment on carbides and phase transition of highly alloyed duplex cast iron. Mater. Des..

[26] Zhou W.B., Zhu H.B., Zheng D.K., Zheng H.X., Hua Q., Zhai Q.J. (2011). Niobium alloying effect in high carbon equivalent grey cast iron. China Foundry.

[27] Chen X.R., Xu J., Hu H., Mohrbacher H., Kang M., Zhang W., Guo A.M., Zhai Q.J. (2017). Effects of niobium addition on microstructure and tensile behavior of as-cast ductile iron. Mater. Sci. Eng. Struct. Mater. Prop. Microstruct. Process..

[28] Prat-Bartés A., Tort-Martorell X., Grima-Cintas P., Pozueta-Fernández L., Solé-Vidal I., Cataluña U.P.d. (2004). Métodos Estadísticos.

[29] Cofiño-Villar A., Alvarez-Antolin J.F., Asensio-Lozano J. (2018). Enhanced Fracture Strength in the Working Layer of Rolls Manufactured in Ni-Hard Cast Iron Alloyed with Mo, Nb and Mg. Metals.

[30] Elliott R. (1988). Cast Iron Technology.

